# Managing an infodemic in the global south: a systematic evidence synthesis from COVID-19 social listening reports

**DOI:** 10.3389/fpubh.2026.1787026

**Published:** 2026-08-26

**Authors:** H. M. V. E. Combrink, P. Mkungeka, C. Bhengu, R. Katarya, B. Smart

**Affiliations:** 1 Faculty of Economics and Management Sciences, University of the Free State, Bloemfontein, South Africa; 2 Interdisciplinary Centre for Digital Futures, University of the Free State, Bloemfontein, South Africa; 3 National Department of Health, Pretoria, South Africa; 4 Department of Computer Science and Engineering, Delhi Technological University, Delhi, India; 5 The Centre for Philosophy of Epidemiology, Medicine and Public Health, University of Johannesburg, Johannesburg, South Africa

**Keywords:** computational infodemiology, infodemic management, infodemiology, public health communication, risk communication and community engagement (RCCE), social listening, social media

## Abstract

The COVID-19 pandemic was accompanied by a widespread infodemic characterised by rapidly evolving information, misinformation, and uncertainty, particularly within low- and middle-income country contexts. Social listening emerged as a critical tool for risk communication and community engagement by enabling real-time monitoring of public concerns, misinformation, and information gaps. This study presents a systematic evidence synthesis of national COVID-19 social listening reports produced in South Africa between 2021 and 2023 to inform infodemic management in the Global South. A total of 91 publicly available social listening reports were identified through coordinated national repositories and screened according to predefined inclusion and exclusion criteria. Following PRISMA 2020 guidance for transparent reporting, misinformation signals and corresponding recommended actions were extracted, thematically coded, and synthesised. The synthesis identified 964 instances of misinformation across 12 thematic categories, with dominant themes relating to COVID-19 disease management, vaccine safety, side effects, and conspiracy narratives. In parallel, 573 recommended action items were consolidated into eight thematic response domains, emphasising community-based engagement, targeted health education, communication strategy development, political and public figure credibility, and continuous monitoring and research. Building on these findings, the study proposes a circular social listening health priority nexus that operationalises the translation of misinformation signals into adaptive public health communication responses. While this synthesis does not evaluate the effectiveness of implemented interventions, it provides structured evidence to support decision-making and priority setting for infodemic management. The findings highlight the importance of context-sensitive, adaptive communication strategies and sustained social listening systems to strengthen public health responses to future infodemics in the Global South.

## 1 Background

The emergence of the SARS-COV-2 virus, which caused the coronavirus disease (COVID-19) outbreak in 2019 in China, led to global hysteria in early 2020, specifically to the development of misinformation around airborne outbreaks (1). This outbreak was declared a pandemic by the World Health Organization (WHO) on the 11th of March 2020 (2). Due to the abundance of misinformation, the public management of the COVID-19 pandemic included a combination of epidemiological and information management. Due to this, the WHO advised governments globally to include social media platforms as part of their approach in risk communication and community engagement (RCCE) during pandemic outbreaks, specifically around the management of misinformation (3). RCCE involves the real-time identification, processing and exchange of information, opinions and advice between response teams and communities affected by the health emergency (4). The inclusion of social media on RCCE is beneficial in that governments can gain insights on the kind of information the public searches for while facilitating information exchange between governments and the public (5). Unfortunately, social media can also perpetuate information voids, leading to potential mis/disinformation during health emergencies. Misinformation refers to false or inaccurate information shared without the deliberate intention to deceive, whereas disinformation refers to intentionally false or manipulated information disseminated with the purpose of misleading individuals or groups. Within public health contexts, both misinformation and disinformation contribute to uncertainty, reduced trust in institutions, and confusion surrounding health behaviours and interventions. Thus, social media coupled with uncertainty has the potential for mis/disinformation and political upheaval to thrive, and once they do, they create an infodemic.

An infodemic refers to the proliferation of information (including valid, true, misleading, and false narratives) that come up across digital and physical environments at a rate faster than the ability to verify the information (6). An infodemic occurs when there is an excessive spread of accurate information mixed with information voids and potential mis/disinformation, typically across different streams of communication, including social media (4–6). The rapid increase of social media users during the COVID-19 pandemic brought to the surface the way widespread and harmful health misinformation can be shared and consumed by social media users on a global scale. Health misinformation is information that hinders the spread of accurate information from health experts at times of disease emergencies like epidemics and pandemics (7). This misinformation undermines efforts by governments to fight disease outbreaks because a significant proportion of governments’ limited resources must be redirected towards fighting the misinformation (6, 7). The increased spread of misinformation online, therefore, increases the need for public health communication and strategies for managing health information infodemics (8). The question is thus which health communication strategies are consistently identified across the various COVID-19 pandemic responses, and more specifically, how can these strategies inform future infodemic preparedness and public health communication strategies in South Africa and similar developing country contexts?

### 1.1 Risk communication and community engagement in the global south

In 2020, the WHO published guidelines for RCCE to accommodate the COVID-19 pandemic (5). RCCE very quickly became an established national emergency preparedness plan (NEPP) across multiple countries in the Global South. The NEPP is informed by the International Health Regulations (IHR) established by the WHO in 2005 (9). The IHR provide countries forming part of the WHO, with a legal framework that consists of the countries’ rights and obligations when it comes to handling public health emergencies. These mandate that countries enhance their capabilities in detecting, assessing, notifying, reporting, and responding to public health threats and emergencies (10). In this context, nations are expected to bolster their capacities to address public health emergencies of international concern (11). South Africa’s response to the digital transformation of public health was shaped by its experience in managing a wide range of major health challenges, from infectious disease outbreaks to pandemics, and by its implementation of the IHR, which required robust information management.

Given the evolving landscape of public health communication that now includes social media, it is crucial to adopt a more proactive strategy for managing health infodemics because of the information flow and access to information brought about by technologies in the digital age. It must be noted that the internet penetration of South Africa is not 100%, and there are parts of the country where digital and social media are not applicable. Despite this, the RCCE technical working group was formed in South Africa to regulate COVID-19 related communication (both digital and physical media like radio stations, newspaper outlets and community engagement) as part of the country’s response during the pandemic. Social listening and infodemic management form part of RCCE core capacities. In South Africa RCCE established a regular feedback mechanism through a social listening working group which focussed on understanding community health concerns (5). RCCE encompasses five core capacities namely, risk communication systems, internal and partner coordination, public communication, community engagement, and addressing uncertainty, perceptions and managing misinformation (12). RCCE incorporates social listening and infodemic management as part of its core strategies (13). Since 2021, social listening played a crucial role in South Africa by providing regular feedback to the South African National Department of Health (NDoH), and provincial health communicators on recommendations for health communication, public health experts, and healthcare workers (5, 14, 15). The group’s responsibilities included supporting various levels of health workers (primary, secondary and tertiary), promoting community engagement, and monitoring online trends. These activities were in response to health messages disseminated across various platforms, including social media, digital media, call centres, group email services, WhatsApp chat groups, media, and opinion articles sent out to the public surrounding these issues and concerns. The group’s efforts were instrumental in ensuring the effective communication of health messages, thereby promoting proactive health behaviours among the general population. Additionally, the social listening team focused on key trends, on concerns and questions from the public, this also included misconceptions, mis/disinformation, and myths (5). The South African social listening working group produced social listening reports using the WHO Africa infodemic response alliance (AIRA) guidelines (5, 16). These reports were compiled by various organisations such as universities, health partners, public institutions and health communicators (17, 18).

### 1.2 Social listening and infodemic management

Social listening as a methodological approach to digital RCCE infodemiology operates in three stages: identification, intervention, and dissemination. Through applying social listening community queries, knowledge voids, and mistrust in health systems are discovered and then shared in a social listening report (19). A social listening report is an instrument that contains the identification of community concerns and questions; the promotion and understanding of risk and health expert advice; building resilience to information; and engaging and empowering communities to take positive action. In this context, the social listening report provides a snapshot of online discourse around a specific infodemic from the information people are sharing through to the recommendations on how to address these concerns.

During 2021, the infodemic was large with rampant misinformation surrounding the COVID-19 vaccine, but as time continued, other health-related issues such as non-COVID-19 disease outbreaks, the emergence of new drugs, health marketing campaigns, and religious influence on public health started surfacing more rapidly on social media (20–23). Thus, a need arose to focus on social listening based on health priorities to inform social listening. In South Africa, the COVID-19 pandemic unfolded within a complex communication environment characterised by high social media usage, unequal access to healthcare information, varying levels of digital literacy, and widespread public uncertainty surrounding vaccines, prevention measures, and government interventions. Like many developing country contexts, the pandemic response was further complicated by the rapid spread of misinformation across digital platforms, community networks, and informal communication channels. In response, the NDoH, together with RCCE partners and stakeholders, implemented social listening mechanisms to monitor public concerns, misinformation narratives, behavioural trends, and information gaps in real time. These RCCE social listening reports formed part of a broader communication surveillance strategy aimed at supporting evidence-based public health messaging, identifying emerging rumours and concerns, and improving communication responsiveness during different stages of the pandemic (21, 22). Additionally, all social listening action items and targeted messaging were translated into all 12 official South African languages, as targeted messaging for RCCE in South Africa.

Health priorities are an important element of health practices within countries to identify health issues. These issues can be identified by health organisations and governments (24). Health priorities can be identified and determined by several factors such as the prevalence and severity of health issues and conditions a country might face as well as the needs of the community in terms of health. In South Africa the National Health Research Committee (NHRC) is tasked with developing health priorities in research to ensure that future research is focused on urgent health priorities (25, 26). These priorities are identified through considering multiple factors such as the burden of the disease, costs of implementing interventions, health needs of communities, etc. However, given the infodemic between 2021 and 2023, there is a lack of research surrounding the health priorities to manage infodemics in South Africa. Therefore, the purpose of this study was to systematically analyse and synthesise recurring misinformation themes, public concerns, behavioural narratives, and health communication priorities identified within the 2021 to 2023 South African RCCE social listening reports to inform future infodemic preparedness and public health communication strategies.

## 2 Methodology

### 2.1 Sources of information

Although this study relied on secondary data sources, the use of publicly available South African RCCE social listening reports provided a structured and longitudinal dataset for analysing recurring misinformation themes, public concerns, and communication priorities during the COVID-19 pandemic. The RCCE social listening reports were developed as part of the NDoH RCCE response and integrated data from multiple digital and community-facing sources, including social media platforms, online news media, WhatsApp monitoring streams, community feedback mechanisms, and public discourse monitoring processes. The reports were compiled by analysing and interpreting the raw data into actionable insights into social listening reports (18).^1^ These reports were used operationally during the pandemic to identify emerging rumours, misinformation trends, behavioural concerns, vaccine hesitancy narratives, and information gaps requiring communication intervention. Rather than extracting information from multiple heterogeneous databases, the study focused specifically on official RCCE social listening reports published between 2021 and 2023 to ensure contextual consistency and reduce variability in reporting approaches. The reports further provided a standardised and recurring national communication surveillance mechanism, enabling longitudinal assessment of misinformation and public concerns across different stages of the pandemic response. A representative from the NDoH RCCE working group assisted in identifying and consolidating all publicly available reports (*n* = 91) included in the study. The study applied a structured review and synthesis approach to consolidate findings across reports while minimising potential extraction bias. The systematic review protocol was registered in the PROSPERO database (CRD42024538254). Ethics approval was granted by the Health Sciences Research Ethics Committee (UFS-HSD2020/1846/2601–0003). The study followed the Preferred Reporting Items for Systematic Reviews and Meta-Analyses Protocols (PRISMA-P) guidance and adhered to the PRISMA 2020 reporting standards for systematic reviews (27). The consolidated reports are publicly accessible (18), while the extracted and curated materials are available within an open repository for public access and reproducibility (17).

### 2.2 Inclusion and exclusion criteria

**Figure 1:**
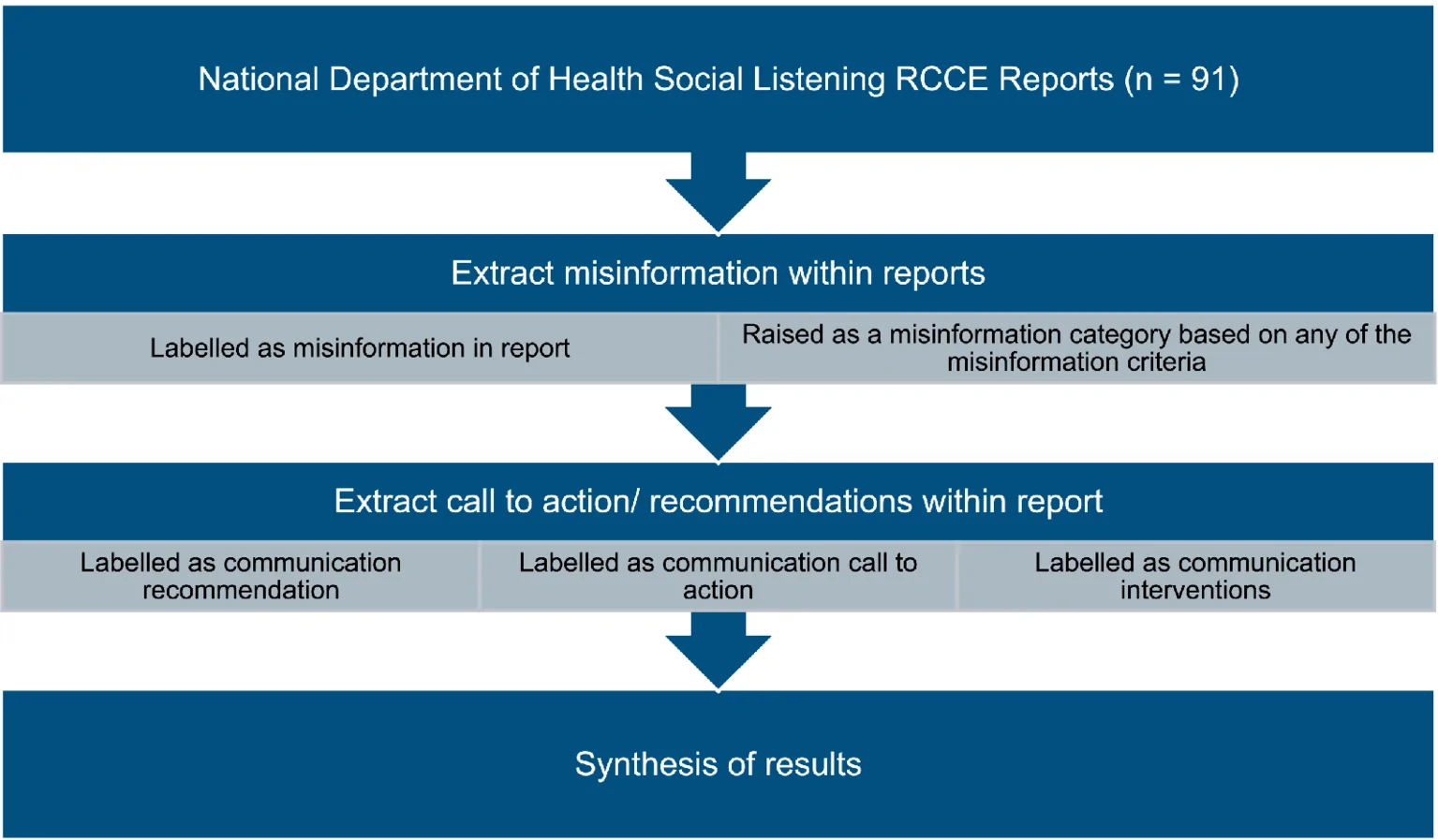
Inclusion and exclusion criteria for the social listening reports.

The social listening report included: the key trends, misinformation, proposed action for RCCE, and methodology and collaboration. The two sections of interest were the misinformation and the proposed actions for RCCE. Studies and reports that were based on the infodemic management criteria, but that did not form part of the South African RCCE group, were excluded. Reports not produced through comprehensive coordination efforts involving multiple organisations were also excluded from the review, as there were three that formed part of these criteria. Only the misinformation and proposed actions sections were considered for the review, and no additional data were included in the study (Figure 1).

### 2.3 Risk of bias assessment and data extraction

The assessment of risk of bias of each article was assessed by using adapted criteria from the Risk of Bias (RoB 2) tool (28) independently by two of the investigators. At least two reviewers performed data extraction independently. All data were extracted to an online form and categorized according to the themes identified by the authors. Reviewers who formed part of the study aided in the systemic classification of the various datapoints extracted. Duplication of ideas, recommendations, and misinformation were removed. In cases where any of these categories were similar, a decision was made by the authors to combine the themes. Collated themes did have an indicator attached to them in the dataset to indicate what the original terms were that became the aggregated theme. This means that a dataset was created for the misinformation categories, and a dataset was created for the strategies that accompanied the misinformation. Once completed, two investigators determined the eligibility for inclusion into the final datasets. These datasets were then imported into a centralised online datasheet, upon which the proposed actions were matched to the misinformation (based on the reports for that specific report). The variables therefore consisted of the date, the report number, the misinformation category, the proposed action that matched that specific misinformation category.

### 2.4 Synthesis of results

The synthesis of results was conducted by matching the proposed actions to the identified misinformation categories. This was done to create a consolidated framework that aligns each type of misinformation with a corresponding proposed action. Each misinformation category identified in the social listening reports was thematically coded and paired with a proposed action from the same report. This pairing was based on the relevance and effectiveness of the action in addressing the specific type of misinformation. The matching was done independently by two investigators to ensure objectivity and reduce bias. The final synthesised dataset was then reviewed and approved by the study authors. It is available and accessible to anyone with an internet connection (18). The synthesis process adhered to standards of systematic review methodology, ensuring the reliability and validity of the results. Once completed, the frequency of the misinformation as well as the proposed actions were determined and the framework for the proposed social listening report was generated.

## 3 Results

### 3.1 Misinformation findings

Within the social listening reports, a total of 964 misinformation instances were recorded from the dataset, totalling 12 different themes (Table 1).

**Table 1:** Misinformation themes extracted from the social listening reports.

Item	Theme	Number of times instance was reported by theme
1	COVID-19 general	119 (12.34%)
2	COVID-19 misinformation	532 (55.19%)
3	COVID-19 vaccination	62 (6.43%)
4	Electronic chip misinformation	6 (0.62%)
5	General information and resources	2 (0.21%)
6	General misinformation	74 (7.68%)
7	Healthcare misinformation	31 (3.22%)
8	Logistics	4 (0.41%)
9	Media misinformation	4 (0.41%)
10	Social factors	2 (0.21%)
11	Vaccination misinformation	126 (13.07%)
12	Community misinformation	2 (0.21%)
	Total	964 (100%)

The first theme COVID-19 general (*n* = 119) included misinformation specific around the COVID-19 disease itself. Subthemes included acceptance of the disease into society (*n* = 56), access to treatment (*n* = 1), general misconceptions (*n* = 1), fraud (*n* = 16), general information and available resources on COVID-19 (*n* = 38), isolation and mental health (*n* = 1), political issues and misinformation (*n* = 4) and misinformation surrounding COVID-19 statistics (*n* = 2). The general themes identified are supported by scholars who have reported on similar trends associated with general COVID-19 information and concerns raised by the public.

The theme specific to COVID-19 misinformation (*n* = 532), was based on the implementation of the management of COVID-19 specific to South Africa. Within this theme, subthemes included rollout and management misinformation (*n* = 4), false case reporting (*n* = 3), conspiracy theories and misinformation including that COVID-19 is embedded with technology (*n* = 14), variant misinformation (*n* = 13), side effects of COVID-19 (*n* = 249), general vaccine misinformation around the global efficacy of vaccines (*n* = 120), and COVID-19 variant misinformation specific in South Africa (*n* = 13). These findings are supported by studies that have explored some of the general COVID-19 misinformation related to the disease. Another group of information within this subtheme mentioned various at home and pharmaceutical remedies that could cure COVID-19, alongside claims that the South African government was withholding these treatments from the population.

The theme of COVID-19 vaccine (*n* = 62) included South African issues of vaccine access (*n* = 17), vaccine hesitancy (*n* = 21), the vaccine mandate and vaccine logistics (*n* = 10), vaccine misinformation and vaccine rollout misinformation (*n* = 4). In the context of vaccine hesitancy and conspiracy theories related to the COVID-19 vaccine, the themes were consistent with the findings from scholars that have investigated this specifically for South Africa. The theme of electronic chip (*n* = 6) pushed the false narrative that the COVID-19 vaccine was embedded with an electronic chip as a means to label the population. General misinformation resources (*n* = 2) included the falsification of official data surrounding the management and hotlines available to the public. General misinformation (*n* = 74) was based around the management of health during the pandemic and included a variety of measures taken by the South African government to manage the pandemic including subthemes around religion, public figures, lockdowns and disease prevention (*n* = 27). Additionally, general health misinformation (*n* = 44) included other issues such as HIV, cholera and other public health concerns raised by the general public according to the reports. Healthcare misinformation (*n* = 31) included misinformation surrounding healthcare workers, as well as information spread by healthcare workers themselves that caused public concern. This included communication from prominent healthcare figures who were reported to the Health Professions Council of South Africa (HPCSA) for medical misconduct. Vaccine misinformation (*n* = 126) included misinformation surrounding side effects (*n* = 66), the vaccine mandate and its implications in the short and long term (*n* = 20), general vaccine logistics and youth vaccination (*n* = 11).

The prevalence of the outlined misinformation from these themes suggests a significant level of uncertainty and confusion among the population about COVID-19, its management, and the vaccine. This could be due to a lack of clear communication, distrust in authorities, or the rapid spread of unverified information. The specific concerns raised, such as vaccine hesitancy and conspiracy theories, indicate a need for targeted public health messaging to address these issues. The mention of remedies and treatments withheld by the government also suggests a level of mistrust in the authorities.

### 3.2 Action items

A total of 573 action items and actionable recommendations were outlined in the social listening reports, which were grouped into six different themes (Table 2).

**Table 2:** Action item themes extracted from the social listening reports.

Item	Theme	Number of times theme was reported
1	Community-based participation, engagement and organisations	148 (25.83%)
2	Education and knowledge building	97 (16.93%)
3	Political buy-in	10 (1.75%)
4	Initiatives for communication, development and governance	299 (52.18%)
5	Logistics and Preventative materials	6 (1.04%)
6	Monitoring and research	13 (2.27%)
	Total	573 (100%)

The first theme centred around community-based participation, engagement and partner organisations that are supporting the NDoH in RCCE efforts (*n* = 148). The types of interventions recommended within the reports included community engagement (*n* = 140), vaccination communication research to improve general public understanding (*n* = 3), and healthcare interventions in communities through public healthcare efforts (*n* = 4). General education and knowledge building (*n* = 97) included information sessions with communities (*n* = 37) to strengthen community understanding, misinformation behavioural change interventions (*n* = 24), and various disease-related information to assist public health efforts such as clean water, general sanitation, and food management practices to prevent public health concerns (*n* = 32).

Political buy-in and public figure engagement (*n* = 10) was a primary recommendation as officials entering into communities and addressing community concerns with credible information was recommended. Another important behavioural change strategy that featured strongly related to initiatives for communication, development and strengthening of governance and management (*n* = 299). These included ways of communicating to the public in the form of conduct (*n* = 70), the types of communication framing that is needed around the vaccine (*n* = 80), how to improve general health communication (*n* = 48), and innovation strategies (*n* = 19) for effective communication using public healthcare and local government, as well as local media to support effective communication. Logistics and preventative materials (*n* = 6) included effective means to manage communication and public concerns around these issues. Finally, general monitoring and research (*n* = 13) was recommended to strengthen public healthcare efforts in the management, communication and acceptance of the general public, especially in the face of the various concerns raised by the public. Action items outline the importance of community engagement, public health education, and knowledge building in managing disease including COVID-19. This includes a focus on interventions such as vaccination communication research and public healthcare efforts. It also underscores the need for political credibility, public figure engagement, and effective communication strategies to address public concerns and misinformation. However, as has been reported across different studies, public trust in the government within South Africa is low. This means that the actionable implementation of interventions needs to contain the most accurate information by managing public concerns. This requires effective communication strategies, including the right framing of messages about vaccines and general health. The action items also outline a need to leverage local media and government resources for effective communication. This includes logistics and preventative measures, and continuous monitoring and research to strengthen public healthcare efforts. The mention of political uptake and public figure engagement indicates the necessity of support from political figures and leaders in these efforts, despite the level of public mistrust in the local and national government.

## 4 Discussion

### 4.1 Evidence synthesis and infodemic response translation

Rather than isolated or static misinformation events, the 964 misinformation instances reveal a persistent and structured pattern of uncertainty, concentrated around disease management, vaccine safety, side effects, and conspiracy narratives. These themes were not evenly distributed but clustered around issues with direct behavioural consequences, particularly vaccine uptake and healthcare-seeking behaviour, indicating higher potential for public health harm.

Across the reviewed reports, misinformation signals were consistently accompanied by proposed response mechanisms, suggesting that social listening was operationalised not merely as a monitoring tool but as an input into decision-making for risk communication and community engagement. The consolidation of 573 action items into eight response domains highlights a dominant emphasis on communication system strengthening, rather than on isolated corrective messaging. Initiatives for communication development and governance, alongside community-based engagement, accounted for most recommended actions, reflecting recognition that infodemic management in low- and middle-income contexts requires structural and relational interventions, not only factual rebuttals. The implicit prioritisation of vaccine-related misinformation reflects its elevated potential for harm, given its capacity to influence collective behaviour at scale.

When synthesised jointly, the misinformation themes and action domains reveal a translation pathway from signal detection to adaptive response. High-frequency misinformation related to vaccines and disease management corresponded with recommendations for targeted health education, tailored communication framing, political and public figure engagement, and sustained community participation. This alignment suggests that national social listening outputs implicitly prioritised context-sensitive, trust-mediated communication, particularly in settings characterised by linguistic diversity, uneven digital access, and low institutional trust. The caveat here is that in the production of these reports, multiple stakeholders were involved and the authors caution that single author social listening may polarise viewpoints and not contextualise the local nuance present within different contexts, especially if social listening and the production of reports were conducted by external (outside the local context) experts.

### 4.2 Circular social listening health priority nexus

Importantly, the synthesis indicates that social listening priorities were not fixed. Emerging misinformation narratives such as evolving claims about side effects or governance failures, necessitated iterative adjustment of communication strategies and health priorities. This adaptive dynamic informed the development of the circular social listening health priority nexus, which conceptualises infodemic management as a continuous cycle of monitoring, signal assessment, prioritisation, response design, and re-evaluation.

**Figure 2:**
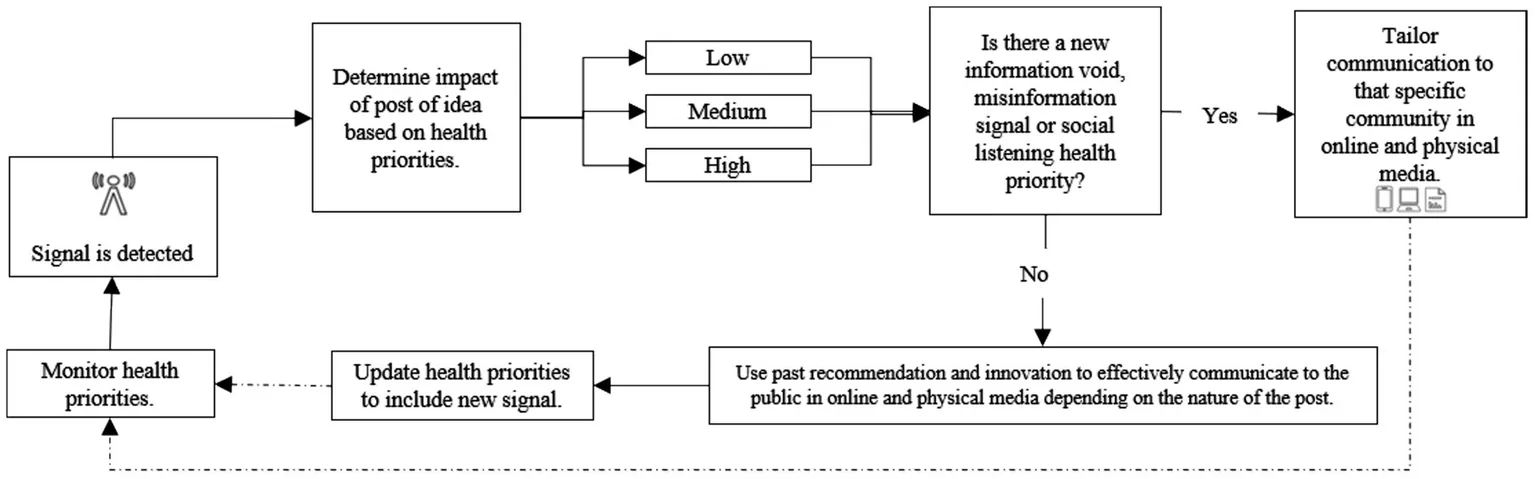
Circular social listening health priority nexus.

Within this nexus, misinformation signals are assessed according to potential harm, reach, and engagement, enabling proportional and timely public health responses. For social listening this was important as online posts were not just in English or geolocated, and language often contained landmarks (reference to specific locations) in a local language dialect (out of the 12 official languages in South Africa). This shows that political uptake and public figure engagement are as crucial for addressing community concerns as much as expert vetting with credible information. Strategies for communication development and strengthening governance and management have also been proposed, including improving general health communication, developing innovative strategies for effective communication, and managing logistics and preventative materials. General education and knowledge building have been emphasised, with recommendations for information sessions with communities, misinformation behavioural change interventions, and the dissemination of various disease-related information to assist public health efforts. Although social listening data are predominantly derived from digital platforms, the resulting insights should not be confined to digital communication strategies. Given persistent inequalities in internet access and digital literacy, social listening outputs should also inform offline communication interventions, including community radio broadcasts, print media, local newspapers, faith-based and community organisation engagement, outreach campaigns, and community health worker activities. Translating digital insights into locally accessible communication channels ensures that priority health messages reach underserved populations and strengthens the equity and effectiveness of public health communication. Continued monitoring and research were also recommended to strengthen public healthcare efforts in communication, management, and public engagement, particularly in response to the evolving concerns raised by communities (Figure 2).

To this end, the social listening priority nexus entails the following procedures to update the health priorities. Firstly, a constant monitoring and evaluation of health priorities determined needs to be performed. This can be in the form of a list of priorities that various organisations use to search for online signals. Next, if a signal is detected the impact of the post or idea based on the health priorities needs to be determined. Based on the review, three categories of harm can be loosely defined as low, medium and high harm. The determination of the harm category is based on buy in, reach, and engagement of a particular post, as outlined in Table 3 (29).

**Table 3:** Harm assessment outline.

Harm assessment	WHO harm characterisation	Operational criteria
Low	Limited potential for infodemic harm	Low reach, low engagement, little or no behavioural adoption; continue monitoring
Medium	Moderate risk of behavioural and community harm	Moderate reach and engagement with emerging behavioural influence; targeted communication
High	Significant risk of public health and institutional harm	High reach, high engagement, evidence of public buy-in or behaviour change with potential health consequences; immediate coordinated response

For example, a post that generates substantial engagement and public acceptance, thereby potentially discouraging people from seeking healthcare or otherwise negatively influencing health behaviours, may be classified as high harm because of the risk it poses. Conversely, a post that attracts little or no engagement or public acceptance may be considered low harm because of the low potential for a widespread infodemic.

Thereafter, it must be determined if this post is a new misinformation signal or if it fits into a current health priority. If the signal fits into a current health priority, then the interventions and known context can be used as a guideline to tailor specific communication to that community or group on physical and/or digital media. If the signal relates to a new type of misinformation or perpetuates a new type of misinformation, then the health priorities need to be updated and a combination of new innovative solutions and experience on effectively managing this type of communication needs to be employed. By synthesising recurring misinformation signals and corresponding action domains across multiple reporting cycles, this review moves beyond descriptive reporting toward structured evidence consolidation. Thereafter, the health priorities need to be updated (in the event of a new signal) and monitoring of new signals must continue. This circular social listening health priority nexus can then be used to continuously monitor potential infodemics and serve as a guideline to manage infodemics. Within the South African context, the findings further reinforce the importance of social listening as a participatory communication mechanism rather than solely a misinformation monitoring tool. This is supported by findings, indicating that effective pandemic communication requires governments and public health structures not only to disseminate information, but also to actively engage with public concerns and perceptions (20, 21). This study has several limitations. Firstly, the review focused exclusively on publicly available South African RCCE social listening reports published between 2021 and 2023. As a result, the exclusion of reports and communication dynamics from 2020 may have influenced the findings, particularly as the early stages of the COVID-19 pandemic were characterised by heightened uncertainty, rapidly evolving scientific information, and the emergence of initial misinformation and conspiracy narratives. Consequently, some early-stage misinformation patterns, behavioural responses, and communication priorities may not have been fully captured within the synthesis. Secondly, the study relied on consolidated RCCE reports rather than raw social media or community-level data. While the reports provided structured and operationally relevant summaries of misinformation trends and public concerns, the synthesis remained dependent on the analytical framing, categorisation approaches, and reporting practices used by the original RCCE reporting teams. Thirdly, the South African context presents considerable linguistic, cultural, and socio-economic diversity, which may not always be fully represented within national-level reporting structures. Although the reports attempted to capture multilingual and geographically diverse communication signals, certain localised narratives and contextual nuances may have been under-represented. Finally, multiple stakeholders contributed to the production and interpretation of the RCCE reports. While this strengthened interdisciplinary interpretation and operational relevance, differences in interpretation, prioritisation, and contextual framing across contributors may have influenced the consolidation of misinformation themes and recommended actions.

## 5 Conclusion

The importance of managing infodemics in physical and digital media is paramount for the digital futures concerning public health. The COVID-19 pandemic has underscored the critical role of accurate information in managing public health crises. The pervasive misinformation, ranging from general misconceptions about the disease to specific misinformation about the vaccine, has significantly impacted public perception and response, leading to issues such as vaccine hesitancy and the spread of conspiracy theories. To combat this, a multi-faceted approach is necessary, encompassing effective community engagement, communication research, communication-related healthcare interventions (such as the creation of digital content, physical media and communicating with the public), and better public health education awareness. However, the low public trust in government within South Africa poses a significant challenge, necessitating the need for cross-party political uptake, public figure engagement, and effective communication strategies. The findings of this study highlight the importance of tailoring interventions to specific population groups, innovating in communication strategies, and continuously monitoring and researching to strengthen public healthcare efforts. Applying findings from digital monitoring to locally trusted communication platforms, e.g., community radio, print and local media, may strenthen essential health information to underserved communities, improving the reach and fairness of public health communication. Ultimately, the management of infodemics requires a nuanced understanding of the evolving landscape of misinformation and public concern. We therefore recommend that social listening, documenting and recording not only misinformation around health, but also the actionable interventions implemented by public healthcare officials are documented and recorded so that systemic interventions for public healthcare may be done effectively to manage an infodemic.

## Data Availability

Publicly available datasets were analyzed in this study. This data can be found here: https://pokello.ufs.ac.za/s/RCCE/page/home.

## Footnotes

1
https://pokello.ufs.ac.za/s/RCCE/faceted-browse/13
